# Theoretical and Experimental Analyses of Nutrient Control in Electrical Conductivity-Based Nutrient Recycling Soilless Culture System

**DOI:** 10.3389/fpls.2021.656403

**Published:** 2021-05-24

**Authors:** Tae In Ahn, Jong Hwa Shin, Jung Eek Son

**Affiliations:** ^1^Department of Agriculture, Forest and Bioresources (Horticultural Science and Biotechnology), Seoul National University, Seoul, South Korea; ^2^Smart Farm Research Center, KIST Gangneung Institute of Natural Products, Gangneung, South Korea; ^3^Department of Horticulture and Breeding, Andong National University, Andong, South Korea; ^4^Research Institute of Agriculture and Life Sciences, Seoul National University, Seoul, South Korea

**Keywords:** Michaelis–Menten equation, sustainability, hydroponics, irrigation, drainage, soilless culture, nutrient recycling

## Abstract

An electrical conductivity (EC)-based closed-loop soilless culture system is practical for in-field deployment. Literature on the closed-loop soilless culture nutrient management premise the limitations in managing recycled nutrients under dynamic changes in individual nutrient uptake concentrations. However, recent systems analysis studies predicting solutions for nutrient fluctuation stabilization in EC-based closed-loop soilless culture systems suggest that the system may have a deterministic side in nutrient variation. This study aims to derive a nutrient control principle in an EC-based nutrient recycling soilless culture system by theoretical and experimental analyses. An integrated model of solutes such as K^+^, Ca^2+^, and Mg^2+^ and water transport in growing media, automated nutrient solution preparation, and nutrient uptake was designed. In the simulation, the intrinsic characteristics of nutrient changes among open-, semi- closed-, and closed-loop soilless cultures were compared, and stochastic simulations for nutrient control were performed in the closed-loop system. Four automated irrigation modules for comparing nutrient changes among the soilless culture systems were constructed in the greenhouse. Sweet pepper plants were used in the experiment. In the experimental analysis, nutrient concentration conversion to the proportion between nutrients revealed distinctive trends of nutrient changes according to the treatment level of drainage recycling. Theoretical and experimental analyses exhibited that nutrient variations in open-, semi- closed-, and closed-loop soilless culture systems can be integrated as a function of nutrient supply to the system’s boundary areas. Furthermore, stochastic simulation analysis indicated that the nutrient ratio in the soilless culture system reveals the nutrient uptake parameter-based deterministic patterns. Thus, the nutrient ratio in the closed-loop soilless culture could be controlled by the long-term feedback of this ratio. We expect that these findings provide theoretical frameworks for systemizing nutrient management techniques in EC-based closed-loop soilless culture systems.

## Introduction

Soilless cropping is attracting attention as one of the principal means for sustainable intensification, which increases yield with minimum adverse environmental impact (Gruda, 2019). The basis for the interest in soilless culture systems is the ease of constructing closed-loop (CL) resource management. However, in practice, the application of CL soilless culture systems is scarce. In South Korea, the proportion of soilless culture occupied by the CL system is only 5% (Lee and Kim, 2019). In Almería, the highest area of soilless production in Spain, only 12% of 3,000 ha soilless cultivation are as uses the drainage reuse system (Massa et al., 2020). On the other hand, the cultivation area of soilless culture continues to increase worldwide (Rodríguez-Delfín, 2012; Walters et al., 2020). For now, the low proportion of the CL system is intertwined with technical constraints. Technological solutions for replacing the open-loop (OL) management practice on the field with the CL system are becoming challenging in soilless culture systems (Massa et al., 2020).

Nutrient management of the recycled nutrient solution is one of the critical barriers in replacing OL nutrient management. Ideally, nutrient management uncertainties can be minimized through the measurement of individual nutrients. Thus, development studies for nutrient management systems using real-time measuring devices, such as ion sensors, have been conducted; however, for a primary nutrient control system replacing OL nutrient management techniques, there were technical constraints (Kläring, 2001; Gieling et al., 2005; Bratov et al., 2010; Lee et al., 2017). The nutrient-sensing technique available at the field level is the electrical conductivity (EC) sensor, representing only the total nutrient concentration. However, nutrient uptake concentration, which complicates nutrient management of the recycled nutrient solution, varies mainly with transpiration and leads to fluctuations in root zone concentrations (van Noordwijk, 1990). Thus, several studies have shown that the CL system could be accompanied by nutrient variation, nutrient imbalance, and subsequent yield loss (Zekki et al., 1996; Kläring, 2001; Hao and Papadopoulos, 2002; Raviv and Lieth, 2008; Miller et al., 2020). Nevertheless, an EC-based system is a promising platform for disseminating nutrient management techniques at the field level. Thus, most of the approaches for managing the recycled nutrients and corresponding adverse effects have been conducted under the EC-based system. These approaches provided various information on nutrient behaviors and crop responses by manipulating the system variables and the parameters, such as the nutrient reuse period (Ehret et al., 2005; Ko et al., 2013), EC control (Rouphael et al., 2016; Signore et al., 2016), semi-closed-loop (SCL) system (level of drainage discharge) (Massa et al., 2011; Rouphael et al., 2016), fertilizer compositions (Hao and Papadopoulos, 2002; Gent and Short, 2012; Neocleous and Savvas, 2015, 2018), and difference in irrigation systems (Zekki et al., 1996; Incrocci et al., 2006; Bouchaaba et al., 2015). In addition to these, Savvas (2002) conducted a study on the control of individual nutrients with 2 weeks intervals of drainage analyses in the laboratory and replenished the adjusted nutrient solutions. Savvas (2002) tested nutrient replenishment methods and discussed their control performance and technical limitations.

However, at the field level, dynamic behaviors in irrigation system variables complicate the understanding and systemizing nutrient control techniques. Transpiration, which impacts nutrient uptake concentration (i.e., the ratio of nutrient to water absorption over time) (van Noordwijk, 1990; Le Bot et al., 1998),subsequently affects drainage volume, leaching fraction, and drainage EC (Ben-Gal et al., 2008; Shin and Son, 2016). Consequently, changes in drainage characteristics influence the recycled nutrient solution (Savvas and Manos, 1999). Understanding nutrient control principles under this condition may require a systems approach. Recently, a theoretical analysis study of a soilless culture system predicted a modified nutrient replenishment method and experimentally verified the theoretical prediction that a modified nutrient replenishment method could stabilize the fluctuations of some individual nutrients in the EC-based CL soilless culture (Ahn and Son, 2019). However, there is still little information on how the individual nutrient could be controlled in the EC-based CL soilless culture so far.

Overall, this study was designed to focus on the nutrient control principle under the irrigation system’s dynamic behaviors and corresponding fluctuations in the nutrient uptake concentration, which affects the nutrient variation characteristics in the soilless culture system.

In this study, theoretical and experimental analyses for nutrient control are based on the following theoretical backgrounds:

(1) A plant’s nutrient uptake phenomenon can be approximated by the Michaelis–Menten equation (Clarkson, 1985; Le Bot et al., 1998; Cott et al., 2018), and a system that follows this mechanism can have a steady-state solution according to the conditions of input variables and parameters (Golicnik, 2011).
(2) Transpiration produces fluctuations in all nutrients’ uptake concentrations in the nutrient solution (van Noordwijk, 1990; Le Bot et al., 1998).

Dynamic changes in nutrient uptake concentration are intrinsically driven by Michaelis–Menten kinetics beneath the transpiration rate variations (Le Bot et al., 1998). On this basis, it can be hypothesized that converting the nutrient concentration to the proportion between nutrients could neutralize the dynamic changes in nutrient uptake concentration and could reveal the nutrient uptake parameter-based deterministic patterns. Consequently, complexity in nutrient control in the EC-based soilless culture system could be expected to be narrowed down to the parameter-based deterministic patterns. This study aims to analyze the nutrient variation patterns according to the degree of nutrient recycling and derive a nutrient control principle in the EC-based CL soilless culture system by constructing an integrated soilless culture system model.

## Materials and Methods

### Automated Irrigation Module Used in This Experiment

**FIGURE 1:**
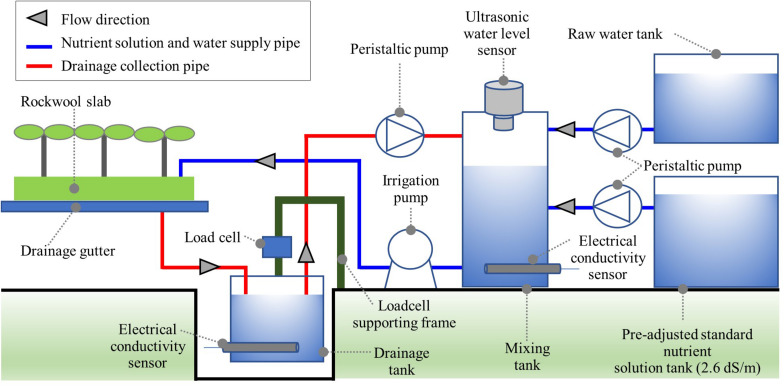
Schematic description of the automated irrigation module for open- (OL), semi-closed (SCL), and closed-loop (CL) soilless culture in the experiment.

The automated irrigation module consisted of a drainage tank, a nutrient solution tank, and a standard nutrient solution tank (Figure 1). In the drainage tank, the weight and EC of the collected drainage were measured by a load cell (JSB-20, CAS, South Korea) and an EC sensor (SCF-01A, DIK, South Korea). In the mixing tank, the nutrient solution’s mixing process was carried out according to the degree of nutrient recycling. The drainage tank’s storage capacity was 11.7 L, and the mixing tank capacity was 19.4 L. Ultrasonic-level sensors (UHA-300, Unics, South Korea) were installed at the top of the mixing tank to monitor the feed amount of drainage, raw water, and standard nutrient solution. Peristaltic pumps (M500, Verderflex, United Kingdom) were used to transfer standard nutrient solutions, drainage, and water from the pre-adjusted standard nutrient solution, drainage, and water tank to the mixing tank. For the irrigation of the mixed nutrient solution, a centrifugal pump (PUN-350M, Wilo Pump, South Korea) was used. A pyranometer (SP-110, Apogee, United States) was used for the integrated solar irradiance for automated irrigation control. A data logger (CR1000, Campbell Scientific, United States) and controller (SDM-CD16AC, Campbell Scientific, United States) were used for measurement, nutrient solution preparation, and irrigation control.

### Greenhouse Experiment of the OL, SCL, and CL Soilless Cultures

The cultivation experiment using the four automated irrigation modules was carried out in a Venlo-type greenhouse located at the experimental farm of Seoul National University (Suwon, South Korea, lat. 37.3°N, long. 127.0°E). The four automated irrigation modules consist of one OL, two SCL, and one CL soilless culture systems. Accordingly, the degree of nutrient recycling was manipulated by applying the OL, SCL, and CL soilless cultures, respectively. The OL soilless culture system uses no drainage in the irrigated solution. The SCL soilless culture system partially uses drainage according to the drainage dilution level. In the CL soilless culture system, most of the collected drainage is reused for the nutrient solution. Basically, we referred to the general process of automated nutrient solution preparation (Savvas and Manos, 1999; Savvas, 2002). The OL irrigation controls the mixing tank EC to the target value. Conventionally, the SCL irrigation reuses the drainage by diluting to a lower EC than the supply EC with water, and stock solutions are applied to the supply EC of the irrigation. However, during the experimental period, the target EC for the irrigated nutrient solution was maintained at 2.6 dS m^–1^. Thus, in the greenhouse experiment, a pre-adjusted standard nutrient solution for the supply EC (2.6 dS m^–1^) was stored in the standard nutrient solution tank, and this was used to replenish the standard nutrient solution. In the greenhouse experiment, the determination of the mixing ratio of drainage, water, and the standard nutrient solution was based on the calculation procedure described in section “Determination of the Mixing Ratio in the Soilless Culture System,” considering the amount of water already added in the pre-adjusted standard nutrient solution.

The four modules of the nutrient recycling degree treatment were composed of OL, CL, and SCL modules with two EC levels for a drainage dilution of 0.65 and 1.3 dS m^–1^ (SCL 0.65 and SCL 1.3). An integrated solar irradiance-based irrigation control was applied. The modules automatically irrigated 150 ml of the nutrient solution prepared in the mixing tank per plant whenever the integrated solar irradiance reaches 100 J cm^–2^. However, the transpiration capacity increases due to plant growth during the cultivation period. Thus, the irrigation frequency or irrigation amount must be adjusted to compensate for the transpiration change. Thus, the supply volume per irrigation event was adjusted by the meteorological condition to compensate for the transpiration capacity change and maintain a drainage ratio of 30%. Each module prepared the nutrient solutions according to the recycling degree before the irrigation event.

Compositions of the standard nutrient solution used in this experiment were 14.56 NO_3_^–^, 1.18 H_2_PO_4_^–^, 3.20 SO_4_^2–^,5.96 K^+^, 9.56 Ca^2+^, 3.38 Mg^2+^, 0.21Na^+^, and 0.30Cl^–^ (in meq L^–1^) as macro-elements and 18 Fe, 10 Zn, 2 Cu, 10 Mn, and 0.5 Mo (in μM) as micro-elements. The target EC of the nutrient solution mixing module was set to 2.6 dS m^–1^, and the EC of the raw water was set to 0.15 dS m^–1^. Daytime temperature, night time temperature, and relative humidity of the greenhouse remained between 25 and 30°C, 15 and 22°C, and 50 and 80%, respectively, by an environmental control system of the greenhouse. Sweet pepper (*Capsicum annuum* L. “Fiesta”) plants were used in the experiment and seeded on July 15, 2011. The sweet pepper plants were transplanted into 90 cm (L) × 15 cm (W) × 7 cm (H) rockwool slabs (Cultilene, Netherlands) on September 29, 2011. Three sweet pepper plants were planted per slab, and three slabs were used per experimental treatment. The planting density was 2.8 plants per square meter.

The sweet pepper plants were cultivated by OL nutrient supply until December 15, 2011, and after that, each treatment was applied, and the experiment was finished on March 9, 2012. On the day before the initiation of the treatments, a large amount of a standard nutrient solution was irrigated in an OL nutrient supply in order to make the initial condition of the nutrients balanced in the rockwool slab of all treatments close to the standard composition.

### Nutrient Analyses and Statistics

To observe the changes in the ratio between nutrients in the root zone of the soilless culture system, samples of nutrient solution in the rockwool slabs were extracted using a syringe. The collection points of the nutrient solution in the rockwool slab were randomly selected for the collection of representative samples of the overall concentration in the root zone. Then, 10 ml of a root zone nutrient solution was collected for each extraction, and this was performed five times to make a 50 ml sample. Six samples per treatment were collected each time every 2 weeks. K^+^, Ca^2+^, and Mg^2+^ were analyzed using an inductively coupled plasma optical emission spectrometer (ICP-730ES, Varian, Australia). The SAS system (version 9.2, SAS Institute, United States) was used for statistical analysis. In the experimental condition, three rockwool slabs were connected to the single automated module system. Thus, the replicate of the sample could have limited data variability. However, the soilless culture using slab-form substrates has an isolated system boundary of the root zone. Thus, even in the single system (i.e., a single greenhouse with a single irrigation system), each slab condition often shows significant variations in root zone nutrient and water according to the microclimate fluctuations of each slab’s location. Therefore, at least, it can be expected that the rockwool slab replicates could statistically rule out these effects in this experimental condition.

### The Theoretical Model for Soilless Culture Systems

**FIGURE 2:**
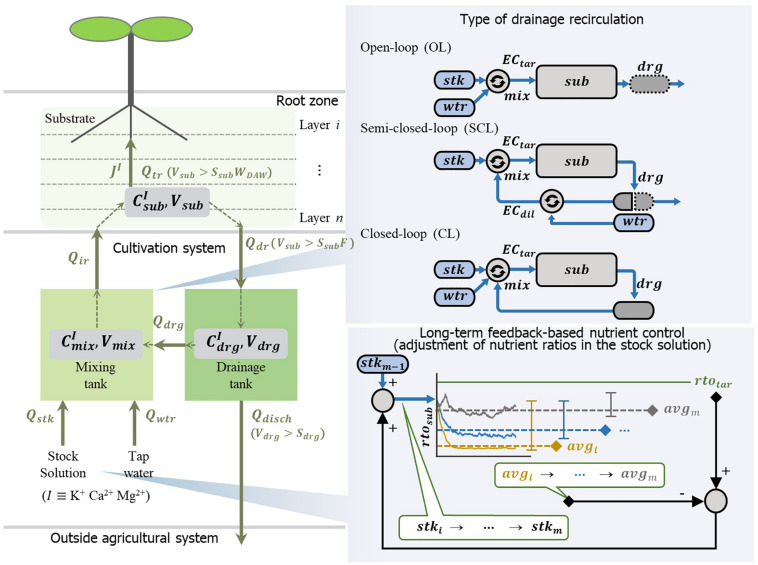
Schematic description of open- (OL), semi-closed- (SCL), and closed-loop (CL) soilless culture systems in the simulation analyses. The water and nutrients are consumed by the transpiration rate (**Q**_**t***r*_) and nutrient uptake rate of the plant (**J^I^**), respectively. Nutrients (**I**) from the stock solution (**Q_s*tk*_**) and tap water (**Q_w*tr*_**) are circulated through the mixing tank, substrate, and drainage tank with the flow rates of irrigation (**Q_i*r*_**), drainage (**Q_d*r*_**), drainage reuse (**Q_d*rg*_**) or discharged (**Q_d*isch*_**) outside the system. OL system supplies the nutrient solution by adjusting the mixing ratio of the stock solution (**s*tk***) and the water (**w*tr***) to the target EC (**E*C*_t*ar*_**). SCL system prepare the drainage reuse by adjusting the mixing ratio of the drainage (**d*rg***) and the water to the dilution EC (**E*C*_d*il*_**). Subsequently, the SCL system adjusts the diluted drainage to the target EC by the stock solution and supplies it to the substrate (**s*ub***). CL system reuses all collected drainage (**d*rg***), and the nutrient solution is prepared by adjusting the mixing ratio of the stock solution (**s*tk***) and water (**w*tr***) to the target EC (**E*C*_t*ar*_**). The long-term feedback-based nutrient control analysis was conducted in the CL system. Whenever the simulation is finished, the difference between the average value (**a*v*g_m_**) of an individual ion ratio in the substrate (**r*t*o_s*ub*_**) and target ratio (**r*t*o_t*ar*_**) was returned to the previous ion ratio of the stock solution (**s*t*k_m−1_**). Subsequently, the modified stock solution (**s*t*k_m_**) was applied to the next simulation.

The models of the soilless culture system used in the simulation consist of water and nutrient transport, nutrient uptake, and nutrient solution mixing (Figure 2). This model’s basic structures were constructed based on previous models of the soilless culture system (Silberbush and Ben-Asher, 2001; Silberbush et al., 2005). The simulation model reflected the automated nutrient solution mixing process of the EC-based soilless culture system. For nutrients and water transport in the substrate, mass flow and diffusion models in porous media were applied (Shackelford and Daniel, 1991; Corwin et al., 1993; Snape et al., 1995). The nutrient uptake by plants follows the Michaelis–Menten equation. The rate of nutrient uptake is mainly driven by concentration. In this study, a modified Michaelis–Menten equation having an additional variable for the transpiration rate was used to reflect more stochastic changes in absorption parameter variations such as plant growth. This model considers reducing the nutrient depletion zone around the roots due to the accelerated mass flow generated by an increase in transpiration rate with plant growth (Sago et al., 2011; Nomiyama et al., 2013). Since transpiration is a variable that includes parameters for plant growth changes (Baille et al., 1994; Ta et al., 2012), the model was used to reflect the change in the nutrient absorption rate with plants’ growth. The definitions, values, units, and sources of the models’ parameters are summarized in Table 1.

**TABLE 1:** Parameters and nomenclature used for the simulations of soilless cultures.

Parameter	Definition	Value	Unit	Source
*J${}_{m\,a\,x}^{K}$*	Ion flux parameter	1.89	10**^–^**^3^meq/plant/min	Estimated from the field experiment data
*J${}_{m\,a\,x}^{C\,a}$*	Ion flux parameter	1.60	10**^–^**^4^meq/plant/min	
*J${}_{m\,a\,x}^{M\,g}$*	Ion flux parameter	1.61	10**^–^**^4^meq/plant/min	
*K${}_{m}^{K}$*	Ion flux parameter	9.00	10**^–^**^3^meq/cm^3^	
*K${}_{m}^{C\,a}$*	Ion flux parameter	2.44	10**^–^**^4^meq/cm^3^	
*K${}_{m}^{M\,g}$*	Ion flux parameter	1.33	10**^–^**^3^meq/cm^3^	
*D*^*K*^	Diffusion coefficient (K^+^)	117.6	10**^–^**^5^ cm^2^/min	Lide, 2005
*D*^*Ca*^	Diffusion coefficient (Ca^2+^)	47.4	10**^–^**^5^ cm^2^/min	Lide, 2005
*D*^*Mg*^	Diffusion coefficient (Mg^2+^)	42.6	10**^–^**^5^ cm^2^/min	Lide, 2005
*W*_*DAW*_	Difficult available water of the substrate	0.0068	Dimensionless	Dubský and Šrámek, 2009
*F*	Field capacity of the substrate	0.74	Dimensionless	Dubský and Šrámek, 2009
*A*	Cross-sectional area of the substrate	630	cm^2^	
*S*_*drg*_	Volume of drainage tank	11,700	cm^3^	
*S*_*sub*_	Volume of substrate layer n	472.5	cm^3^	
*C*	Miliequivalent ion concentration		meq cm**^–^**^3^	
*Q*	Water flow rate		cm^3^ min**^–^**^1^	
*V*	Volume of water		cm^3^	
θ	Water content		Dimensionless	
*J*	Nutrient uptake rate		meq/plant/min	
*EC*	Electrical conductivity		dS m**^–^**^1^	
*OL*	Open-loop			
*SCL*	Semi-closed-loop			
*CL*	Closed-loop			
Subscript				
*drg*	Relative to drainage			
*sub*	Relative to substrate			
*mix*	Relative to mixing tank			
*stk*	Relative to a stock solution			
*wtr*	Relative to raw water			
*tar*	Relative to a target value			
*dil*	Relative to dilution value			
*n*	Relative to the substrate layer			
*i*	Relative to starting number			
*m*	Relative to the number of simulation			
*ir*	Relative to irrigation			
*tr*	Relative to transpiration			
Superscript				
*I*	Relative to a type of ion			

Several assumptions were made to reduce the complexity of the simulation:

(1) Total ion concentration is in a linear relationship with EC in the nutrient solution (Savvas and Adamidis, 1999). Based on this assumption, the EC variable in the automated nutrient solution mixing process was replaced by the total equivalent concentrations in nutrient solution mixing simulation.
(2) The target nutrients selected for the simulation were K^+^, Ca^2+^, and Mg^2+^, major cations in macronutrients (Marschner and Marschner, 2012).
(3) This simulation aims to analyze the effect of nutrient uptake concentration on nutrient management in the soilless culture system; thus, the interaction between nutrients and changes in the nutrient uptake parameters were not reflected.
(4) The nutrient ratio used in this study indicates the proportion between the milliequivalention concentrations (C^*I*^/(C^*K*+^ + C^*Ca2*+^ + C^*Mg2*+^); I ≡ K^+^,Ca^2+^, Mg^2+^; C is the milliequivalent ion concentration).

### Water Transport in the Soilless Culture System

Water transport in a CL soilless culture system occurs between the substrate, the drainage tank, and the mixing tank, and these correspond to Eqs. 1–3, respectively.

(1) $$\frac{d\,V_{s\,u\,b,n}}{d\,t}=\left\{ \begin{array}{c} Q_{i\,r}-Q_{d\,r,1}-Q_{t\,r,1},n\,1\\ Q_{d\,r,n-1}-Q_{d\,r,n}-Q_{t\,r,n},a\,n\,d\,n\,1 \end{array}\right.$$

(2) $$\frac{d\,V_{d\,r\,g}}{d\,t}=Q_{d\,r,n}-Q_{d\,r\,g}-Q_{d\,i\,s\,c\,h}$$

(3) $$\frac{d\,V_{m\,i\,x}}{d\,t}=Q_{d\,r\,g}+Q_{s\,t\,k}+Q_{w\,t\,r}-Q_{i\,r}$$

where *V*(cm^3^) is the volume of water, subscript *sub*, *n* is the water content of each layer when the substrate is divided into *n* layers, *drg* is the drainage tank, and *mix* is the mixing tank. *Q*(cm^3^ min^–1^) is the flow rate of the water; subscript *ir* is the irrigation flow rate to the top of the substrate (*n* = 1);subscripts *dr*,*n*-1 and *dr*,*n* are the flow rates of the drain from the *n* - 1 layer and the *n* + 1 layer, respectively; and *tr*,*n* represents the transpiration rate of the substrate layer *n*. Here, *Q*_*dr*_,*_*n*_* is the difference between the irrigation rate to the substrate and the transpiration rate (*Q*_*ir*_ – *Q*_*tr*,1_or *Q*_*dr*_,*_*n*_*_–1_ – *Q*_*tr*_,*_*n*_*) (Shin et al., 2014). However, *Q*_*dr*_,*_*n*_* and *Q*_*tr*_,*_*n*_* are restricted by the field capacity (*F*, dimensionless) and difficult available water (*W*_*DAW*_, dimensionless), respectively, for the volume of the layer of the substrate (*S*_*sub*_, cm^3^). And *Q*_*dr*_,*_*n*_* flows only when *V*_*sub*_,*_*n*_* > *S_*sub*_F*, and *Q*_*tr*_,*_*n*_* flows only when *V*_*sub*_,*_*n*_* > *S_*sub*_W_*DAW*_*. *Q*_*drg*_, *Q*_*stk*_, and *Q*_*wtr*_ correspond to the drain, stock solution, and raw water flow rates, respectively, introduced into the mixing tank when irrigation to the root zone is required. *Q*_*disch*_ is a variable that is applied to the SCL system, meaning the flow rate of the nutrient solution discharged to the outside when the volume of stored drainage (*V*_*drg*_) exceeds the capacity of the drainage tank (*S*_*drg*_, cm^3^).

### Nutrient Transport and Uptake Models in the Soilless Culture System

In a soilless culture system, the movement of nutrients follows the same path as water, and the movement of nutrients between the substrate, drainage tank, and mixing tank can be expressed as Eqs. 4–6, respectively.

(4) $$V_{s\,u\,b,n}\,\frac{d\,C_{s\,u\,b,n}^{I}}{d\,t}=\{\begin{array}{c} \begin{array}{c} Q_{i\,r}\,C_{m\,i\,x}^{I}-Q_{d\,r,1}\,C_{s\,u\,b,1}^{I}-J_{1}^{I}-\\ \frac{D^{I}\,A\,\left(\theta_{1}\,C_{s\,u\,b,1}^{I}-\theta_{n+1}\,C_{s\,u\,b,n+1}^{I}\right)}{Z},n=1\\ Q_{d\,r,n-1}\,C_{s\,u\,b,n-1}^{I}-Q_{d\,r,n}\,C_{s\,u\,b,n}^{I}-J_{n}^{I}-\frac{D^{I}\,A\,\left(\theta_{n}\,C_{s\,u\,b,n}^{I}-\theta_{n+1}\,C_{s\,u\,b,n+1}^{I}\right)}{Z}\\ -\frac{D^{I}\,A\,\left(\theta_{n}\,C_{s\,u\,b,n}^{I}-\theta_{n-1}\,C_{s\,u\,b,n-1}^{I}\right)}{Z}\\ ,n>1 \end{array} \end{array}$$

(5) $$V_{d\,r\,g}\,\frac{d\,C_{d\,r\,g}^{I}}{d\,t}=Q_{d\,r,n}\,C_{s\,u\,b,n}^{I}-Q_{d\,r\,g}\,C_{d\,r\,g}^{I}-Q_{d\,i\,s\,c\,h}\,C_{d\,r\,g}^{I}$$

(6) $$V_{m\,i\,x}\,\frac{d\,C_{m\,i\,x}^{I}}{d\,t}=Q_{d\,r\,g}\,C_{d\,r\,g}^{I}+Q_{s\,t\,k}\,C_{s\,t\,k}^{I}-Q_{i\,r}\,C_{m\,i\,x}^{I}+Q_{w\,t\,r}\,C_{w\,t\,r}^{I}$$

where *C*(meq cm^–3^) represents the equivalent concentration of nutrients and superscript *I* is the type of ions (K^+^, Ca^2+^, and Mg^2+^). Subscripts *sub*, *drg*, *mix*, *stk*, and *wtr* represent the source of the nutrients and indicate substrate, drainage tank, mixing tank, stock solution, and raw water, respectively. *J*^*I*^(meq plant^–1^ min^–1^) means the uptake rate of nutrients. The final term of Eq. 4 refers to the diffusion of solutes in a substrate, where *D*(10^–5^ cm^2^ min^–1^) is the diffusion coefficient of an ion *I*, *A*(cm^2^) is the cross-sectional area of the substrate; θ_*n*_ is the water content of the *n* layer (*V*_*sub*_,*_*n*_*/*S*_*sub*_, dimensionless); *Z*(cm) is the height of the *n* layer. In the last layer, the diffusion term from *n* + 1 is excluded because the diffusion occurs from the previous layer only.

The Michaelis–Menten equation was used to model the nutrient uptake rate of the plant (Eq. 7).

(7) $$J_{n}^{I}=\frac{J_{m\,a\,x}^{I}\,C_{s\,u\,b,n}^{I}}{K_{m}^{I}+C_{s\,u\,b,n}^{I}}$$

where $J_{max}^{I}$ means the maximum absorption rate of the ion and $K_{m}^{I}$ is the Michaelis–Menten constant. $J_{n}^{I}$ is the absorption rate of individual nutrient *I* in layer *n*, and the equation applying the change in the rate of absorption of nutrients to the increase of transpiration rate (*Q*_*tr*_,*_*n*_*) is shown in Eq. 8.

(8) $$J_{n}^{I}=\frac{J_{m\,a\,x}^{I}\,C_{s\,u\,b,n}^{I}\,Q_{t\,r,n}}{K_{m}^{I}+C_{s\,u\,b,n}^{I}\,Q_{t\,r,n}}$$

### Determination of the Mixing Ratio in the Soilless Culture System

In the simulation, the degree of nutrient recycling was manipulated by applying nutrient solution mixing processes of the OL, SCL, and CL soilless cultures. The nutrient solution preparation process for the OL, SCL, and CL soilless cultures of the above section of the greenhouse experiment of the OL, SCL, and CL soilless cultures was converted to this study’s soilless culture system model.

*Q*_*stk*_, *Q*_*drg*_, and *Q*_*wtr*_ of the SCL soilless culture system are determined by Eqs. 9–11, respectively.

(9) $$Q_{s\,t\,k}=\left\{ \begin{array}{c} Q_{i\,r}-Q_{w\,t\,r}-Q_{d\,r\,j},i\,f\,V_{d\,r\,g}>0\\ \frac{Q_{i\,r}\,\left(C_{t\,a\,r}-\sum C_{w\,t\,r}^{I}\right)}{\sum C_{s\,t\,k}^{I}-\sum C_{w\,t\,r}^{I}},i\,f\,V_{d\,r\,g}\leq 0 \end{array}\right.$$

(10) $$Q_{d\,r\,g}=\left\{ \begin{array}{c} \frac{Q_{i\,r}\,\left(C_{d\,i\,l}\,\sum C_{s\,t\,k}^{I}-\sum C_{s\,t\,k}^{I}\,\sum C_{w\,t\,r}^{I}-C_{d\,i\,l}\,C_{t\,a\,r}+C_{t\,a\,r}\,\sum C_{w\,t\,r}^{I}\right)}{\sum C_{s\,t\,k}^{I}\,\sum C_{d\,r\,g}^{I}-\sum C_{s\,t\,k}^{I}\,\sum C_{w\,t\,r}^{I}-C_{d\,i\,l}\,\sum C_{d\,r\,g}^{I}+C_{d\,i\,l}\,\sum C_{w\,t\,r}^{I}},\\ i\,f\,V_{d\,r\,g}>0\\ 0,i\,f\,V_{d\,r\,g}\leq 0 \end{array}\right.$$

(11) $$Q_{w\,t\,r}=\left\{ \begin{array}{c} \frac{Q_{d\,r\,g}\,\sum C_{d\,r\,g}^{I}-Q_{d\,r\,g}\,C_{d\,i\,l}}{C_{d\,i\,l}-\sum C_{w\,t\,r}^{I}},i\,f\,V_{d\,r\,g}>0\\ Q_{i\,r}-Q_{s\,t\,k},i\,f\,V_{d\,r\,g}\leq 0 \end{array}\right.$$

where *C*_*dil*_(meq cm^–3^) is the equivalent target concentration for the dilution of the total nutrients in the drainage and *C*_*tar*_ is the target equivalent concentration for the total nutrients in the nutrient solution to be supplied to the plant. The OL system corresponds to the case when *V*_*drg*_ ≤ 0 in Eqs. 9–11.

*Q*_*stk*_, *Q*_*wtr*_, and *Q*_*drg*_ for the CL system are determined by Eqs. 12–14, respectively.

(12) $$Q_{s\,t\,k}=\frac{Q_{i\,r}\,\left(C_{t\,a\,r}-\sum C_{w\,t\,r}^{I}\right)-Q_{d\,r\,g}\,\left(\sum C_{d\,r\,g}^{I}-\sum C_{w\,t\,r}^{I}\right)}{\sum C_{s\,t\,k}^{I}-\sum C_{w\,t\,r}^{I}}$$

(13) $$Q_{w\,t\,r}=Q_{i\,r}-Q_{d\,r\,g}-Q_{s\,t\,k}$$

(14) $$Q_{d\,r\,g}=Q_{d\,r,n}$$

### Parameter Estimation and Theoretical Analysis of the Nutrient Variations

The CL soilless culture system discharges no drainage. Water replenishment into the system follows the transpiration amount in the system. Nutrients removed from the system correspond to the absorbed nutrients by plants. Thus, the Michaelis–Menten parameters of the nutrient uptake model were estimated by performing a numerical integration-based progress curve analysis using the water and nutrient inputs, transpiration, and nutrient concentration data measured in the CL soilless culture experiment. Simulation and progress curve analysis of these models were performed using Berkeley Madonna 8.3.23 (Berkeley Madonna, Inc., University of California, Berkeley).

The estimated nutrient uptake model parameters were applied to the OL, SCL, and CL soilless culture system models. We performed two simulation analyses as follows:

(1) According to the degree of nutrient recycling, namely, OL, SCL, and CL irrigation models, the nutrient ratio changes were analyzed. The nutrient ratio changes were investigated concerning the degree of nutrient supply into the OL, SCL, and CL systems’ boundaries.
(2) This study hypothesizes that converting the nutrient concentration to the nutrient ratio between nutrients could neutralize the dynamic changes in nutrient uptake concentrations and reveal the nutrient uptake parameter-based deterministic patterns. On this basis, a simulation scenario was applied for the derivation of the long-term feedback-based nutrient control technique.

Under the simulation scenario, the transpiration rate, one of the main disturbance factors for nutrient uptake concentration, was manipulated by a random walk. The long-term feedback nutrient control was performed by changing the nutrient ratios in the stock solution for nutrient replenishment. The feedback period was set to 12 weeks, which is the total period of the simulation. After each simulation, the difference between the target nutrient ratio and average nutrient ratio (target average) was fed back to adjust the stock solution’s nutrient ratio (Figure 2). Thus, as an example, if the average nutrient ratio is higher than the target ratio, negative feedback is returned to the stock solution’s nutrient ratio.

## Results and Discussion

### Soilless Culture System Model Verification

**FIGURE 3:**
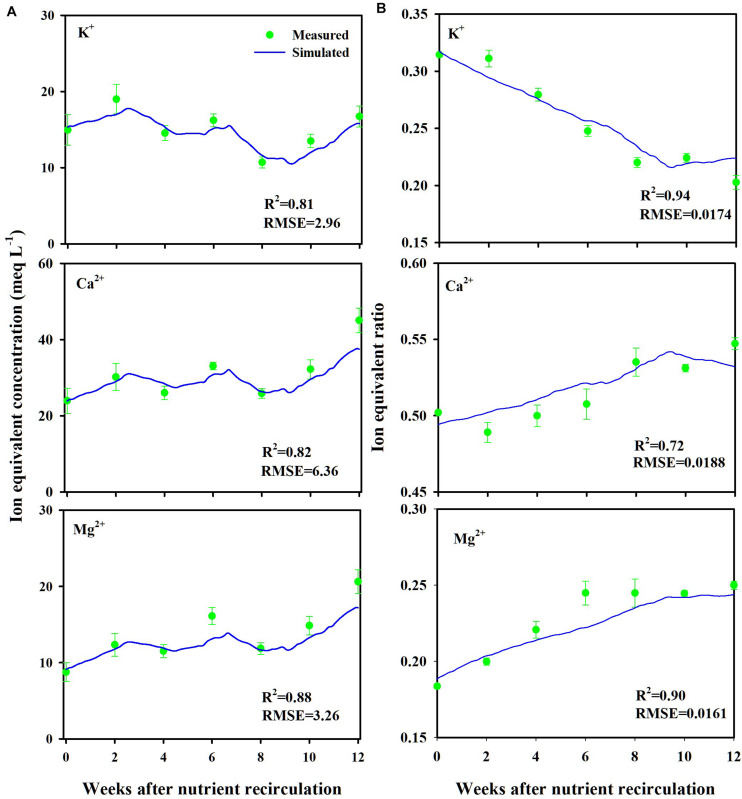
Measured (mean ± SD) and simulated ion equivalent concentrations **(A)** and ion equivalent ratios **(B)** in the substrates of the closed-loop soilless culture system (CL). Ion equivalent concentrations of the substrates represent ion concentration in the whole layers.

**FIGURE 4:**
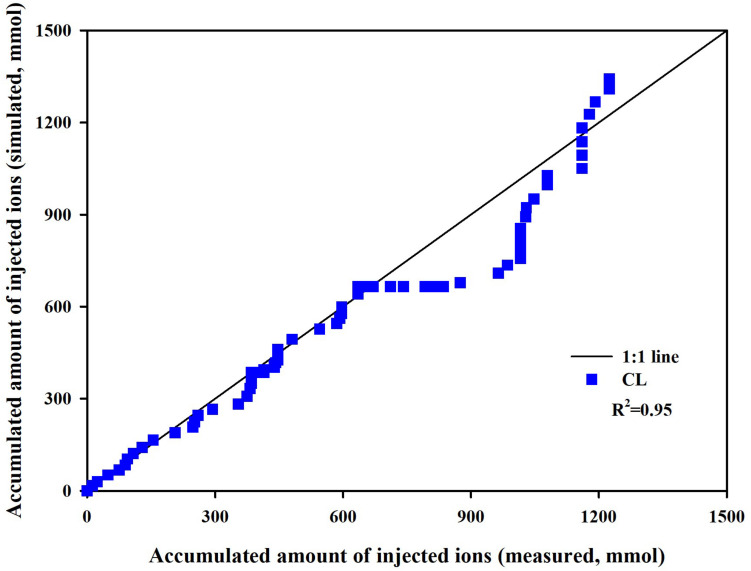
Measured versus simulated amounts of total ions supplied to the closed-loop soilless culture system (CL).

Measured and simulated nutrient concentrations and ion ratios in the CL substrate showed close agreements with *R*^2^ values of 0.81–0.88 and a root mean square error (RMSE) of 2.96–6.36 meq L^–1^ (Figure 3A) and *R*^2^ values of 0.72–0.94 and an RMSE of 0.0161–0.0188 (dimensionless) (Figure 3B), respectively. Measured and simulated values in all nutrient concentrations fluctuated with similar patterns. However, the nutrient ratio of the CL showed distinguishable directional trends. The cumulative nutrient supply to the simulated and experimental CL systems showed good agreement with the measured data with an *R*^2^-value of 0.95 (Figure 4).

Overall, the soilless culture system model closely simulated the nutrient concentration variations in the CL soilless culture. Furthermore, the nutrient supply into the CL soilless culture system’s boundary was simulated with a determination coefficient of 0.95. The replenished nutrients in a CL soilless culture system have a relation with the absorbed nutrient and water in the system (Savvas, 2002). Nutrient concentration variations observed in the CL soilless culture system represent the combined effect of nutrient and water absorption, nutrient replenishment, irrigation, and drainage recycling. Thus, the nutrient preparation process and nutrient variation aspects in the soilless culture system were verified here. However, it needs to be noted that the simulation model estimated a relatively high *K${}_{m}^{K}$*. This might be derived from the current model’s structural limitations. The modified nutrient uptake model applies transpiration variations to the nutrient uptake parameter variations. Thus, the parameter estimation could be valid in the simulation condition.

### Changes in Nutrient Concentration and Ratio in the Experiment

**FIGURE 5:**
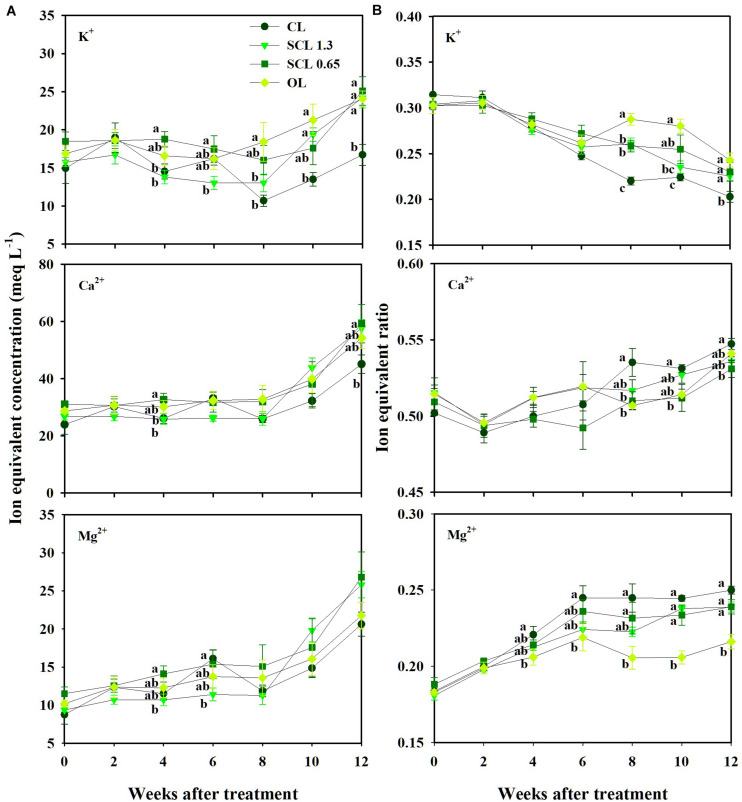
Changes in the ion equivalent concentration **(A)** and ion equivalent ratios **(B)** in the substrate of the closed-loop (CL), semi-closed-loop (SCL 1.3 and SCL 0.65), and open-loop (OL) soilless culture system, respectively. SCL 1.3 means an EC level for a drainage dilution of 1.3 dS m^–1^. Data represent the mean ± SD from three rockwool slabs of each treatment. Significant differences (*P* < 0.05) between treatments are indicated by different letters.

The nutrient concentration in the substrate indicated similar trends throughout the experiment period. The concentration variations were not distinct from the treatment and showed little consistency in the treatments’ significant differences (Figure 5A). On the other hand, when the nutrients were converted to a ratio between the nutrients, the nutrient variation trends indicated more distinguishable trends in individual nutrients and each treatment (Figure 5B). The ratio of K^+^ in CL, which has the least nutrient supply, was significantly lower than that of the other treatments and deviated most from the initial value. OL, which has the highest nutrient input, was the closest to the initial ratios. In the CL treatment, Mg^2+^ and Ca^2+^ remained higher than those in other treatments after observing significant differences 4 weeks after treatment. Mg^2+^ and Ca^2+^were significantly lower in OL and tended to be the closest to the initial ratios (Figure 5B). Thus, unlike nutrient concentrations, the nutrient ratio conversion revealed increasing or decreasing individual nutrients under the nutrient concentration’s overall fluctuation.

Furthermore, those increasing or decreasing trends in nutrient ratios and K^+^ leveled off 6 weeks after treatment. These results suggest that the nutrient ratios have more deterministic aspects than the nutrient concentration. On the other hand, the nutrient concentrations were relatively difficult to distinguish between the nutrients. The concentration trends between the treatments also showed complicated fluctuations. Changes in nutrient concentrations in the root zone in soilless culture systems vary depending on the difference between irrigated nutrient concentration and nutrient uptake concentration (van Noordwijk, 1990). The nutrient uptake concentration represents the ratio of nutrients to water taken up over time (Le Bot et al., 1998). Thus, the change in the uptake concentration results from variations in the transpiration and nutrient uptake rate. The transpiration could affect mass flow near the root system (Marschner and Marschner, 2012). The transpiration could also partially drive the uptake rate of ions like Ca^2+^ (Adams and Ho, 1993; Mengel, 2009). Thus, an ion-specific interaction between transpiration and nutrient uptake could affect the substrate’s nutrient concentration variations.

**FIGURE 6:**
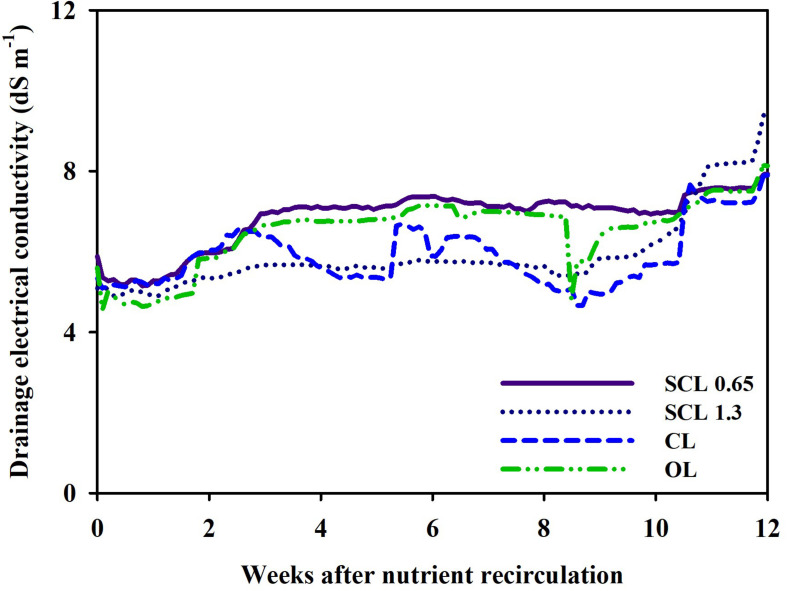
Changes in electrical conductivity (EC) in the drainage tank of the closed-loop (CL), semi-closed-loop (SCL 1.3 and SCL 0.65), and open-loop (OL) soilless culture system, respectively.

However, in this study, the concentration of the substrate nutrient showed the synchronized fluctuations. As explained above, the nutrient concentration indicated similar trends and showed an overall gradual increase from the 8 weeks onwards. The drainage EC of CL, SCL, and OL systems followed similar tendencies (Figure 6). However, the CL system’s drainage EC showed more sensitive variations to the nutrient concentration change. In contrast, in SCL and OL, which have relatively low drainage reuse proportions, less sensitive EC changes were observed. The low drainage reuse leads to the accumulation of residual drainage in the tank, and subsequently, the EC in the drainage tank responds less sensitively to the substrate nutrient concentration changes. As a result, depending on each system’s storage conditions, the sensitivity of the EC fluctuation in the drainage tank was different, but the overall trends indicated similarity with variations in the substrate nutrient concentration. The overall increase in the nutrient concentration 8 weeks after treatment could mainly result from the increased transpiration rate as plant growth progresses. In general, a plant’s transpiration rate is accelerated as the plant expands its leaf area during growth (Bailey et al., 1993). In this study, the transpiration gradually increased to the end of the greenhouse experiment (Figure 8A). This may suggest that the change in transpiration has a more dominant global effect on the nutrient concentration changes in nutrient concentrations.

Changes in transpiration and consequent variations in nutrient absorption concentration mainly drove the macroscopic fluctuations in the overall nutrients and EC. On the other hand, nutrient absorption is mainly dominated by transporters’ arrangement located on the root surface (Arsova et al., 2020). Thus, converting the nutrient concentrations to nutrient ratios might have reduced the global effect of transpiration on the nutrient concentrations and revealed the distinguishable directional trends. Subsequently, it can be conjectured that the nutrient ratio changes in a soilless culture system mainly reflect the nutrient uptake phenomenon driven by the nutrient transporter. That is, the nutrient ratio changes could implicate the Michalis–Menten-driven nutrient uptake. Consequently, the nutrient ratio filtered the variations in nutrient uptake concentrations variations and showed the combined effect of nutrient recycling degree and nutrient uptake parameters.

### Theoretical Analysis of Nutrient Ratio in the OL, SCL, and CL Soilless Cultures

**FIGURE 7:**
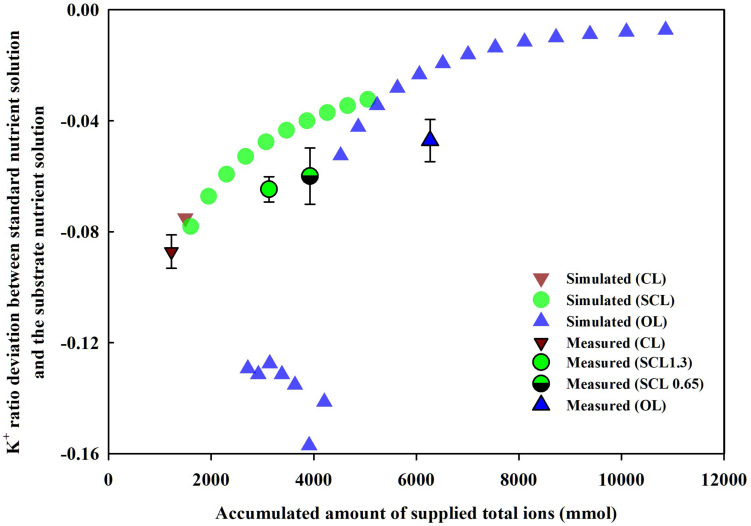
Changes in the measured (mean ± SD) or simulated K^+^ ratio deviation between standard nutrient solution and the substrate nutrient solution by supplied amounts of total ions of the soilless culture systems at the end of the experiment and the simulation. CL, SCL, and OL mean the closed-loop, semi-closed-loop, and open-loop soilless culture systems, respectively. SCL 1.3 means an EC level for a drainage dilution of 1.3 dS m^–1^.

Simulation (1), described in section “Parameter Estimation and Theoretical Analysis of the Nutrient Variations,” showed the nutrient ratio changes according to the degree of nutrient recycling, namely, OL, SCL, and CL irrigation models. The degree of nutrient recycling is also strongly related to the degree of nutrient supply into the system boundaries of OL, SCL, and CL systems. Thus, the nutrient ratio changes of the soilless culture systems were summarized on the basis of a nutrient supply (Figure 7). As a representative, when the K^+^ ratios in the soilless culture systems were summarized for nutrient inputs, specific trends were observed. As nutrient supply increases, i.e., as the degree of nutrient recycling decreases, the nutrient ratio’s deviation from the standard ratio approached zero. Furthermore, the K^+^ ratios in the experiment showed similar trends as the simulation. The deviation between the nutrient ratio measured in the experiment and the standard ratio decreased in the order of CL, SCL 1.3, SCL 0.65, and OL.

**FIGURE 8:**
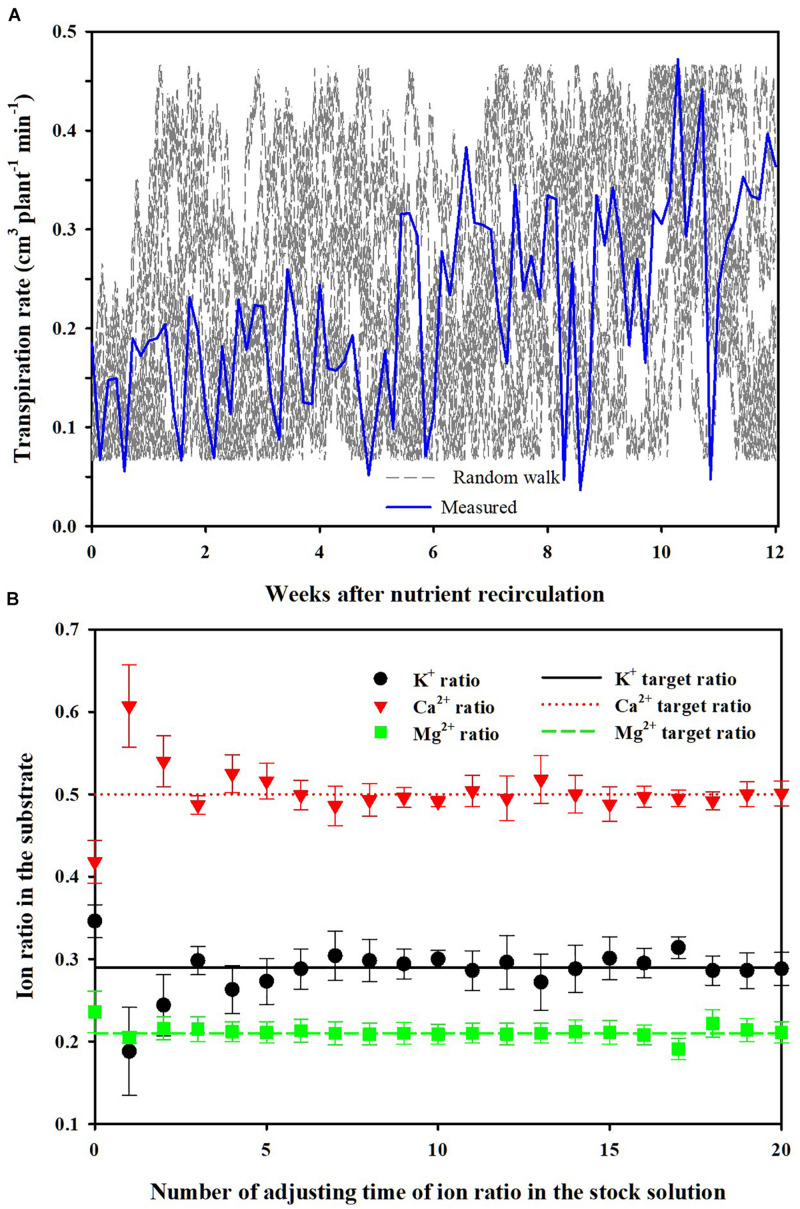
Random walk transpiration rates considering disturbance in the uptake concentrations by simulation and measured transpiration rates in the experiments **(A)** and changes in ion ratios (mean ± SD) in the substrate according to the number of adjusting the time of ion ratios in the stock solution under the random walk disturbance **(B)**.

The nutrient variations in the CL soilless culture system are often considered a black box in some way for its dynamic behaviors in uptake concentration (van Noordwijk, 1990). Thus, in CL soilless culture, managing the nutrient uptake concentration effect is often approached as empirical methods. The literature on the EC-based CL soilless culture system investigated the appropriate interval or level of nutrient solution renewal (Ehret et al., 2005; Ko et al., 2014), drainage discharge (Massa et al., 2011), and EC control (Rouphael et al., 2016; Signore et al., 2016). The CL soilless culture system was featured due to uncertainties in nutrient management. However, Simulation (1) shows that although the OL, SCL, and CL soilless culture systems have been separated from each other, the systems’ nutrient ratios can be summarized continuously based on the systems’ nutrient inputs.

In Simulation (1) of section “Parameter Estimation and Theoretical Analysis of the Nutrient Variations,” a modified concept on the nutrient variations of OL, SCL, and CL soilless culture systems was presented. The nutrient ratio changes of each system were macroscopically integrated into nutrient inputs to the systems. In this respect, the soilless culture models could be simplified by the inflow and the outflow into the system boundary and the system’s uptake rate (Eq. 15). Moreover, with these critical parameters, the theoretical framework for the nutrient variations of OL, SCL, and CL soilless culture systems could be reconstructed.

(15) $$V_{s\,y\,s}\,\frac{d\,C_{s\,y\,s}^{I}}{d\,t}=Q_{s\,t\,k}\,C_{s\,t\,k}^{I}+Q_{w\,t\,r}\,C_{w\,t\,r}^{I}-\frac{J_{m\,a\,x}^{I}\,C_{s\,y\,s}^{I}}{K_{m}^{I}+C_{s\,y\,s}^{I}}-Q_{o\,u\,t}\,C_{s\,y\,s}^{I}$$

where *Q*_*stk*_ and *Q*_*wtr*_ are the flow rates of the stock nutrient solution and raw water flowing into the boundary of the soilless culture system, respectively, and *Q*_*out*_ is the flow rate of the nutrient solution discharged outside the boundary. $C_{sys}^{I}$ corresponds to the concentration of a nutrient in the system. $C_{stk}^{I}$ and $C_{wtr}^{I}$ are concentrations of a nutrient in the stock solution and raw water, respectively. *V*_*sys*_ is the volume of nutrient solution in the system. *V*_*sys*_ in Eq. 15 cannot exceed the system’s capacity, and the consumed water is replenished repeatedly and thus is assumed to be constant.

In the SCL system, the boundary of the system includes the mixing tank and the drainage tank because the drainage is partially circulated or discharged outside the system. Therefore, *Q*_*stk*_ and *Q*_*wtr*_ correspond to the supply rate of nutrients and water to the soilless culture system, respectively; and *Q*_*out*_ in Eq. 15 corresponds to the flow rate of the nutrient solution discharged from the drainage tank (*Q*_*disch*_ in Eq. 5). In the case of the CL system, the boundary is the same as that of the SCL system, but *Q*_*out*_ does not occur, and the steady-state solution, in this case, can be summarized as in Eq. 16.

(16) $$C_{s\,y\,s}^{I}=\frac{K_{m}\,C_{s\,t\,k}^{I}\,Q_{s\,t\,k}+K_{m}^{I}\,C_{w\,t\,r}^{I}\,Q_{w\,t\,r}}{J_{m\,a\,x}^{I}-C_{s\,t\,k}^{I}\,Q_{s\,t\,k}-C_{w\,t\,r}^{I}\,Q_{w\,t\,r}}\,\left(16\right)$$

This indicates that the point of convergence for $C_{sys}^{I}$ is determined according to the concentrations of the supplied stock solution ($C_{stk}^{I}$) and raw water ($C_{wtr}^{I}$) and their inflow rates (*Q*_*stk*_ and *Q*_*wtr*_, respectively) and means that when the inflow rates exceed the maximum nutrient uptake rate of the plant ($J_{m\,a\,x}^{I})$, the system becomes unstable. However, in the EC-based CL soilless culture system, *Q*_*stk*_ is determined based on the total equivalent concentration of nutrients in the system; as a result, the feeding rate of the total nutrients follows the uptake rate of total nutrients at a certain level. Thus, too excessive increases or decreases can be controlled by general EC control practice. Under this condition, the nutrient ratio conversion could reveal the parameter-based nutrient variations. Unlike dynamic variations of nutrient concentration, the parameter-based nutrient variations could indicate deterministic changes. Thus, these deterministic changes could be controllable by long-term feedback.

Assuming that the $C_{sys}^{I}$ is the total equivalent concentration of all nutrients in the system measured by EC and $C_{sys}^{I}$ is controlled in steady-state through EC feedback, Eq. 16 theoretically predicts that $C_{sys}^{I}$ converges to a constant value. This means that the individual nutrient variations could also level off to steady-state. Additionally, the terms including *Q*_*wtr*_ in Eq. 16 may contribute to the system fluctuation by replenishing the water consumed through transpiration. Thus, its influence is determined by the mineral concentration in the raw water. However, in most CL or SCL soilless culture studies, including the cultivation experiment of this study, and OL soilless culture, irregular fluctuations in the total nutrient concentrations relative to its initial values have been reported often (Hao and Papadopoulos, 2002; Massa et al., 2011; Shin and Son, 2016; Signore et al., 2016; Lee et al., 2017; Moon et al., 2018, 2019).

However, the recent literature theoretically deduced a modified nutrient replenishment method for steady-state management of EC and experimentally demonstrated its effect (Ahn and Son, 2019). Here, this study’s theoretical prediction on the effect of steady-state EC control was observed in the greenhouse experiment. When the total ion concentration was controlled to the initial target value, the individual nutrients of the EC-based CL soilless culture system rapidly converged into the average steady state.

Overall, Simulation (1) defines that the SCL and OL soilless culture systems are mainly driven by the nutrient input and discharge. On the other hand, the nutrient variation in a CL soilless culture system is mainly determined by the relationship between nutrient inputs and nutrient uptake parameters.

### Nutrient Ratio Control in the CL Soilless Culture

Simulation (2) shows the average changes of the nutrient ratios and their standard deviations over the simulation period when performing the nutrient balance control described in section “Parameter Estimation and Theoretical Analysis of the Nutrient Variations.” For each simulation, randomized transpiration rates were generated by the random walk method (Figure 8A). The mean values of K^+^, Ca^2+^, and Mg^2+^ ratios gradually converged to the control target values as the times of stock solution adjustment increased (Figure 8B). Also, the standard deviations gradually decreased. Even though the random walk generated arbitrary uptake concentration in every simulation, the nutrient ratios in the CL soilless culture system gradually approached the target ratios. Thus, every soilless culture system simulated in this scenario has experienced different nutrient uptake concentrations.

In this simulation, a different path of the transpiration by random walk was applied every time, within the range of maximum fluctuation during the cultivation period. Furthermore, the theoretical and experimental analyses were conducted under the substrate condition, which has a non-homogeneous root-zone condition. These can be regarded as extreme conditions. However, the average nutrient ratio approached the target value even in a long-term feedback period of 12 weeks under various nutrient uptake concentrations. This means that the discussion about Eq. 16 is also theoretically valid. Moreover, other soilless culture systems using no substrate such as nutrient film and deep-flow technique could have less spatial variations in nutrient and water distribution than a substrate-based system. Thus, this theoretical framework can be expected to be compatible with other soilless culture systems. Consequently, the results of the theoretical and experimental analyses suggest that the nutrient variations in EC-based soilless cultures could be managed even under the weekly based long-term nutrient analysis conditions.

In a previous study, nutrient control with a 2 weeks feedback period was conducted in an EC-based CL soilless culture system (Savvas, 2002). Here, the difference between the target concentration and the current concentration at every nutrient analysis event was applied as the feedback value. This corresponds to the proportional control from the viewpoint of control engineering. The proportional control can generate a steady-state error, and the literature reported similar trends in the nutrient concentrations. However, no discussion in this respect was conducted so far. Although the nutrient uptake concentration fluctuated by transpiration could be a short-term phenomenon, the cumulative difference between the supplied nutrient concentration and nutrient uptake concentration could result in a particular trend of nutrient concentration or nutrient ratio changes on a time series. Neutralizing the transpiration effects from the nutrient management in the recirculating system could reveal the kinetic domain of nutrient uptake. Thus, in this domain, nutrient variation complexity could be narrowed down to nutrient uptake parameter aspects.

## Conclusion

This study hypothesized that converting the nutrient concentration to the nutrient ratio could neutralize the dynamic changes in nutrient uptake concentration and reveal the nutrient uptake parameter-based deterministic patterns. The greenhouse experiment of the degree of nutrient recycling, namely, OL, SCL, and CL soilless cultures, showed that the nutrient ratio conversion could provide more distinguishable nutrient behaviors. Thus, the complexity of nutrient uptake concentration in the CL soilless culture was narrowed down to the parameter-based level. The simulation analysis confirmed that the nutrient ratio, which represents the parameter-based deterministic changes, could be controlled by long-term feedback control. Although this study analyzed limited nutrients, the three macro-elements K^+^, Ca^2+^, and Mg^2+^ are ions that account for almost half of EC and total equivalent concentrations in a soilless culture system. We expect that subsequent studies based on the theoretical framework established in this study could be advanced to the extended techniques for the rest of the ions, including the micro-elements. In conclusion, our findings provided theoretical frameworks for systemizing nutrient management techniques and suggested a new nutrient control technique in the EC-based CL soilless culture.

## Data Availability Statement

The original contributions presented in the study are included in the article/supplementary material, further inquiries can be directed to the corresponding author/s.

## Author Contributions

TIA and JES conceived the research and prepared the manuscript. TIA and JHS performed the experiments and analyzed the results. All authors read and approved the final manuscript.

## Conflict of Interest

The authors declare that the research was conducted in the absence of any commercial or financial relationships that could be construed as a potential conflict of interest.
